# Integrating TRPV1 Receptor Function with Capsaicin Psychophysics

**DOI:** 10.1155/2016/1512457

**Published:** 2016-01-14

**Authors:** Gregory Smutzer, Roni K. Devassy

**Affiliations:** Department of Biology, Temple University, 1900 N. 12th Street, Philadelphia, PA 19122, USA

## Abstract

Capsaicin is a naturally occurring vanilloid that causes a hot, pungent sensation in the human oral cavity. This trigeminal stimulus activates TRPV1 receptors and stimulates an influx of cations into sensory cells. TRPV1 receptors function as homotetramers that also respond to heat, proinflammatory substances, lipoxygenase products, resiniferatoxin, endocannabinoids, protons, and peptide toxins. Kinase-mediated phosphorylation of TRPV1 leads to increased sensitivity to both chemical and thermal stimuli. In contrast, desensitization occurs via a calcium-dependent mechanism that results in receptor dephosphorylation. Human psychophysical studies have shown that capsaicin is detected at nanomole amounts and causes desensitization in the oral cavity. Psychophysical studies further indicate that desensitization can be temporarily reversed in the oral cavity if stimulation with capsaicin is resumed at short interstimulus intervals. Pretreatment of lingual epithelium with capsaicin modulates the perception of several primary taste qualities. Also, sweet taste stimuli may decrease the intensity of capsaicin perception in the oral cavity. In addition, capsaicin perception and hedonic responses may be modified by diet. Psychophysical studies with capsaicin are consistent with recent findings that have identified TRPV1 channel modulation by phosphorylation and interactions with membrane inositol phospholipids. Future studies will further clarify the importance of capsaicin and its receptor in human health and nutrition.

## 1. Introduction

The chemosensory properties of capsaicin have been widely examined in the human oral cavity. This review describes how psychophysical studies with capsaicin complement molecular and physiological studies of the capsaicin receptor, Transient Receptor Potential Vanilloid type 1 (TRPV1).

**Figure 1:**
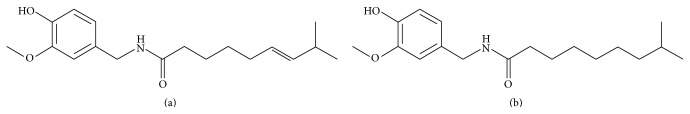
Chemical structure of (a) capsaicin and (b) dihydrocapsaicin (image 1b by Vyacheslav Nasretdinov via Wikimedia Commons).

Capsaicin (8-methyl-*N*-vanillyl-*trans*-6-nonenamide) is a hydrophobic compound that is produced by the plant genus* Capsicum* and gives chili peppers their spicy taste [1]. The chemical structure of capsaicin and the related compound dihydrocapsaicin are shown in Figure 1. These vanilloids act as deterrents against ingestion of the plant by mammals, and as a means to inhibit fungal infections caused by insects [2, 3]. Due to its pharmacological properties, capsaicin is widely used as a topical analgesic to decrease muscle and joint pain [4]. In the human oral cavity, capsaicin is an irritant that produces both thermal (hot) and nociceptive (burning or stinging) sensations [5] by activating neurons of the maxillary and mandibular branches of the trigeminal nerve (Cranial Nerve V) [6]. TRPV1 receptor cells in oral tissue project via the lingual branch of the trigeminal nerve to the trigeminal spinal nucleus, which is also known as the trigeminal nuclear complex of the brain stem [7].

In addition, capsaicin stimulates metabolic activity, promotes negative energy balance through an increase in energy exposure, plays a role in weight control, and increases the oxidation of fatty acids [8–11]. In humans, this vanilloid may also suppress orexigenic (appetite-stimulating) sensations [8]. This compound may also exhibit antitumorigenic properties [12] and may function as a vasodilator that facilitates heat dissipation [13]. Finally, morbidity studies suggest that the consumption of spicy foods that contain chili peppers may increase human longevity [14].

*Transient Receptor Potential Vanilloid Type 1 in the Oral Cavity.* The mandibular branch of the trigeminal nerve provides sensation to the lower third of the face, the anterior two-thirds of the tongue, the oral mucosa, and the lower jaw [6]. Since capsaicin binds to receptors located within trigeminal neurons, sensitivity to this oral stimulant is usually restricted to the anterior two-thirds of the tongue [15].

Capsaicin is an agonist that binds to the TRPV1 receptor [16–19], a well characterized ion channel that localizes to peripheral terminals of primary afferent neurons that sense both pain and heat. TRPV1 is widely expressed in central nervous system (CNS) tissue and highly expressed in sensory neurons of the dorsal root ganglion [19]. This receptor also localizes to neurons that line the oral and nasal cavities [10], where it is found in a subpopulation of sensory afferent nociceptive nerve fibers [20]. The two major trigeminal fiber systems that express functional TRPV1 receptors are the myelinated A_delta_-fibers and the unmyelinated C-fibers [10, 21, 22]. In addition to gustatory tissue, TRPV1 is also expressed in afferent fibers and in keratinocytes of the oral and nasal cavities [8, 17]. Keratinocytes are important in maintaining the integrity of the immune response in skin cells [23].

Within cells, TRPV1 receptors localize to both plasma membranes and internal membranes (such as ER membranes) where this channel mobilizes internal calcium (Ca^2+^) stores [18]. This nonselective cation channel has a tenfold higher preference for Ca^2+^ where it functions as a biosensor of noxious heat and chemical agonists [19]. Activators for this receptor include proinflammatory substances such as 9-hydroxyoctadecadienoic acid, lipoxygenase products, resiniferatoxin from* Euphorbia resinifera* plants, endocannabinoids, capsaicin and its* cis* isomer zucapsaicin, dihydrocapsaicin, protons, and peptide toxins [16, 17, 19, 24, 25]. The alkaloid piperine from black pepper [26] and zingerone (vanillylacetone, commonly known as ginger) may also activate TRPV1 receptors in heterologous systems [27], and in trigeminal ganglia [28]. Gingerol also activates TRPV1 in cultured neurons [29]. In heterologous systems, sodium cyclamate, saccharin, aspartame, and acesulfame potassium may also function as TRPV1 agonists [30]. Finally, TRPV1 channels are also activated by increases in membrane voltage (>+60 mV), and at temperatures with thresholds near 43°C [19].

**Figure 2:**
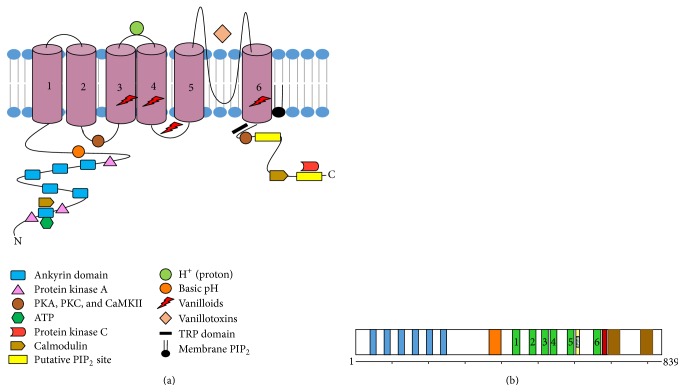
Schematic diagram that illustrates key structural features of TRPV1 control and regulation of channel activity and ion transport. (a) Each TRPV1 subunit is a 95 kDa protein that contains six transmembrane domains, a pore region between the fifth and sixth transmembrane domains, and extended intracellular N- and C-terminal tails. Modified from Bevan et al. [33]. Vanilloid binding site from Yang et al. [32]. (b) Protein domains of canonical TRPV1 receptor (human TRPV1 receptor contains 839 amino acid residues). Blue columns represent N-terminal ankyrin repeats, and orange column represents the cytosolic linker domain. The six numbered green columns represent transmembrane domain regions of the receptor, and light yellow column represents the alpha helical domain of the pore-forming region found between transmembrane domains 5 and 6. The red column represents the cytosolic TRP domain, and the two brown columns represent putative C-terminal PIP_2_ binding domains. Horizontal line represents the linear sequence of amino acids from the N-terminus to the C-terminus. Modified from Liao et al. [43].

At the receptor level, capsaicin binds to the region that spans transmembrane domains 3 to 4 of the TRPV1 receptor [31] (see Figure 2(a)). Bound capsaicin orients in a “tail-up, head-down” configuration where the vanillyl and amide groups form specific interactions that anchor the ligand to the receptor [32]. Amino acids Y512 (helix S3), S513 (helix S3), E571 (S4-S5 linker), and T671 (helix S6) of murine TRPV1 bind the ring of capsaicin via hydrogen bonding while amino acid residue T551 (helix S4) binds the amide group of capsaicin [32]. (Murine Y512 corresponds to Y511 of human TRPV1.) Since ligands bind to the cytosolic side of TRPV1 [18], these agonists must traverse the plasma membrane to access the intracellular ligand-binding site of TRPV1 [19, 33]. One exception is proton activation at pH of <6.0. Below this pH, protons bind to the extracellular outer-pore domain of TRPV1 [33] for activation of homotetrameric TRPV1 channels [34].

Several antagonists have been identified that block TRPV1 function. Ruthenium red (ammoniated ruthenium oxychloride) is a noncompetitive TRPV1 antagonist, and this dye functions as a pore blocker [35]. In addition, the capsaicin analogue capsazepine is a powerful antagonist for human TRPV1 channels [36].

TRPV1 is also an important heat sensor in peripheral sensory neurons [33]. This channel exhibits high sensitivity to heat, with Q10 values of approximately 25 [16, 33]. TRPV1 channel activation leads to the elevation of cytosolic Ca^2+^, and the subsequent release of neuropeptides such as substance P and calcitonin-gene related peptide by exocytosis [37]. Substance P is found in nociceptive sensory fibers that express TRPV1, and in sensory fibers near taste buds of several mammalian species [38]. This neurotransmitter modulates signals for both heat and pain [39]. This undecapeptide is released from neurons following an increase in cytosolic Ca^2+^ levels, which may be mediated by TRPV1 channels. The release of substance P causes an increase in vasodilatation and vascular permeability, which may cause local edema [40]. Finally, this neuropeptide may stimulate mast cells to release inflammatory mediators such as histamine [41].

However, sustained activation of trigeminal neurons by capsaicin depletes presynaptic concentrations of substance P [42]. This depletion can interfere with a variety of sensory functions, including the response of animals to noxious heat stimuli [42].

## 2. Structure and Function of TRPV1 Receptor

The fully assembled TRPV1 receptor is a homotetramer that forms an outwardly rectifying cation channel that is permeable to both monovalent and divalent cations [33]. This channel exhibits single-channel conductance of 50–100 picosiemens [33]. Each TRPV1 subunit contains six transmembrane domains and a hydrophobic pore region between transmembrane domains 5 and 6 [19, 33]. The N and C termini of this receptor both localize to the cytoplasm [19, 33]. A schematic diagram of the structure and regulatory sites of TRPV1 is shown in Figure 2.

At the subunit level, six consecutive ankyrin repeats localize to the cytosolic N terminus, followed by a conserved linker region that connects the last N-terminal ankyrin repeat domain to the pre-S1 helix that contains *α*-helical and *β*-strand secondary structure [43]. This helix is followed by four transmembrane domains (S1–S4), and the S4-S5 linker region. Transmembrane domain 5 (S5) adjoins the hydrophobic pore region, a region that is required for proton activation, heat activation, or activation by chloroform and isoflurane [44]. Transmembrane domain six (S6) links the receptor to its C-terminal cytosolic region, which contains the 25-amino acid long TRP domain [TRP box] [45].

Ankyrin repeats at the cytoplasmic N-terminal domain bind calmodulin and ATP for modulating TRPV1 activation [45, 46]. The N-terminal domain also contains several putative phosphorylation sites that serve as binding sites for both calmodulin and ATP [47] (see Figure 2(a)).

The 150-amino acid residue C-terminus also interacts with cytosolic proteins and ligands. This terminus contains phosphoinositide and calmodulin binding domains, and protein kinase C (PKC) consensus sites [48–51]. This C-terminal cytosolic region of TRPV1 also includes the conserved transient receptor potential (TRP) domain that interacts with the S4-S5 cytoplasmic linker for involvement in channel gating, and binding sites for phosphatidylinositol 4,5-bisphosphate (PIP_2_) [18, 43]. The highly conserved TRP box also interacts with the pre-S1 helix of TRPV1. The TRP box is necessary for allosteric channel activation [52] and may also regulate the formation of functional tetrameric TRPV1 receptors [53].

**Figure 3:**
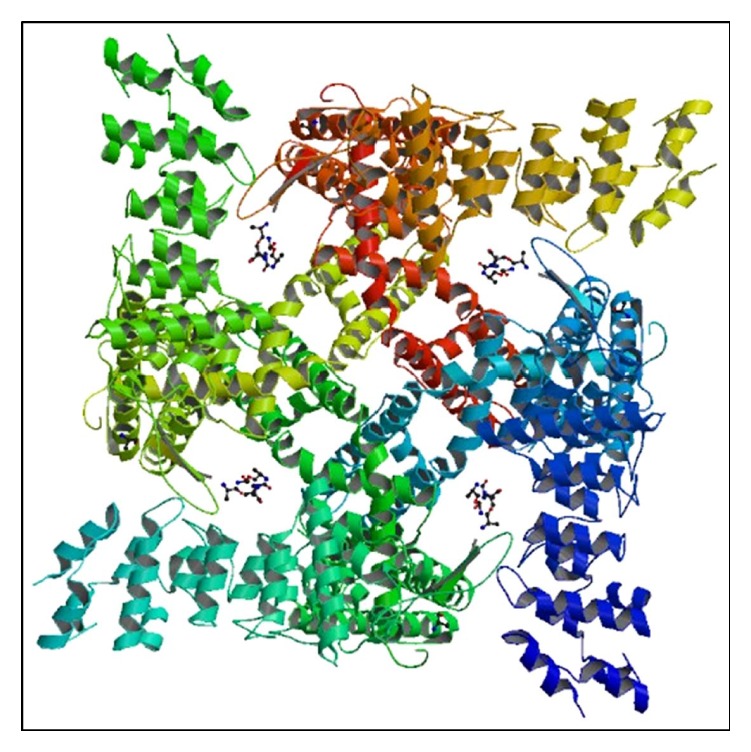
Reconstruction of homotetrameric rodent TRPV1 ion channel complexed with capsaicin that was obtained by single particle electron cryomicroscopy. Image from PDB ID 3J5R. Liao et al. [43] (http://www.rcsb.org/ [112]).

Single particle electron cryomicroscopy (cryo-EM) permits the three-dimensional reconstruction of large protein complexes at high atomic resolution without the need to crystallize the protein [32, 43, 54]. The transmembrane topology and subunit organization of TRPV1 have recently been identified by cryo-EM structures at 4.2 to 4.5 Å resolution [43, 46]. These studies indicate that TRPV1 resembles voltage-gated potassium channels [32] where the symmetrical arrangement of four identical protein subunits forms a centrally located ion-conducting pore [19, 32, 33] (see Figure 3). This pore-forming loop of TRPV1 homotetramers contains an outer-pore turret, a pore helix, and a selectivity filter [55, 56], where each of the four subunits contributes to the ion-conducting pore and selectivity filter [45]. Also, a second intervening pore loop is flanked by Segment 1–Segment 4 voltage-sensor-like domains [43]. See Figure 3.

### 2.1. Mutagenesis Studies of TRPV1

Mutagenesis studies have shown that M547 and T551 of human and rodent TRPV1 are required for sensitivity to capsaicin [57]. In addition, Glu600 and Glu648 (*pK*_a_ of side chain is 4.07) of TRPV1 receptors both face the extracellular surface. These two residues flank the pore-forming region of the receptor and regulate channel activation by extracellular protons [19]. At pH below 6.0, protons open the TRPV1 channel. Above this pH, protons lower the threshold for TRPV1 activation by agonists including capsaicin [19]. Finally, replacing a glutamate with the neutral amino acid glutamine (E600Q) causes a greater than tenfold leftward shift in the capsaicin dose-response curve [58], with EC_50_ values shifting from 520 nM to 40 nM. The physiological characteristics of these TRPV1 E600Q mutant receptors were thought to resemble wild-type channels operating under acidic conditions [58].

Several domains of the TRPV1 receptor are critical for heat sensitivity. In particular, several amino acid residues in the pore region and the C-terminal domains are critical for heat sensitivity [33]. Mutagenesis studies have further shown that I696A, W697A, and R701A in the C-terminal TRP box domain of this receptor resulted in complete loss or reduction of heat sensitivity, capsaicin sensitivity, or voltage activation [59]. In contrast, an F640L mutation in the pore-forming region of this receptor enhanced channel sensitivity to heat and capsaicin but abolished the potentiating effect of extracellular protons [60].

### 2.2. G-Protein-Coupled Receptors and TRPV1 Sensitization

Sensitization (activation) at the receptor level is consistent with psychophysical studies with capsaicin. Sensitization is thought to occur by phosphorylating this receptor, and maximal stimulation (and mobilization to the plasma membrane) of TRPV1 may occur when the receptor is maximally phosphorylated [19]. TRPV1 channels contain multiple phosphorylation sites that stimulate sensitization [35, 51]. Sensitization may arise following phosphorylation of TRPV1 by either PKC (via IP_3_ signaling) or protein kinase A (PKA) (via cyclic AMP signaling). These kinases are both activated by distinct G-protein-coupled receptors [20, 35, 61].

Agonists such as prostaglandin E_2_ and serotonin bind to receptors that activate the stimulatory G-protein G*α*_*s*_. This G-protein subunit then triggers the synthesis of cyclic AMP by adenylate cyclase. The resulting increase in cytosolic cyclic AMP activates PKA, which in turn phosphorylates serine 116 and threonine 370 in the N-terminal region of TRPV1 (see Figure 2(a)). The phosphorylation of serine 116 likely inhibits dephosphorylation of TRPV1 after activation by capsaicin [35]. Finally, PKA activity reduces desensitization of TRPV1 by phosphorylating serine 116 [19], which allows increased sensitivity to the agonist anandamide [62].

TRPV1 also interacts with signal transduction pathways that activate G-protein-coupled receptors such as bradykinin B_2_ and endothelin receptors [19, 63]. These receptors trigger the release of G*α*_q_ subunits from heterotrimeric G-proteins, which in turn activate the enzyme phospholipase C-beta (PLC*β*). PLC*β* then hydrolyzes PIP_2_ to the second messengers inositol 1,4,5-trisphosphate (IP_3_) and diacylglycerol (DAG) [64]. DAG activates PKC*ε*, which in turn phosphorylates TRPV1 receptors at serine and threonine residues [51, 64, 65]. This phosphorylation sensitizes this receptor to heat, protons, or chemical agonists [19]. In general, inflammatory agents enhance TRPV1 activity by phosphorylation pathways that stimulate IP_3_ turnover and enzymatic activation of PKC*ε* [19, 20, 35].

Phosphorylation of serine/threonine residues at both N- and C-termini of TRPV1 receptors by PKC*ε* increases the sensitivity of this channel to both capsaicin and heat by lowering thresholds for these two stimuli [66]. In addition, phosphorylation of serine 800 reverses the desensitization of TRPV1 that occurs from prolonged stimulation with capsaicin, and subsequently increases the sensitivity of this channel to agonists [19].

In addition to serine 800, PKC enhances TRPV1 function by phosphorylating serine 502 [51]. Phosphorylation by PKC at these residues is thought to modulate TRPV1-evoked currents [51], by possibly lowering temperature thresholds for opening TRPV1 channels [35, 51]. Sensitization (potentiation) of TRPV1 by PKC-mediated phosphorylation may also increase SNARE-dependent exocytosis of TRPV1-containing vesicles to the plasma membrane [67]. This mobilization of TRPV1 to the plasma membrane could then increase the overall channel activity of these receptors in sensory cells by increasing access to capsaicin.

In summary, TRPV1 may be most responsive to agonists if the channels are maximally phosphorylated prior to exposure to agonist. Increases in the intensity of capsaicin perception in the oral cavity may mirror greater numbers of phosphorylated TRPV1 receptors, or increased phosphorylation of individual TRPV1 receptors.

### 2.3. TRPV1 Desensitization and Calcium

The repeated exposure of TRPV1 to agonists such as capsaicin fails to activate this receptor or may only minimally activate this receptor. This process is known as desensitization and occurs by a Ca^2+^-dependent mechanism that leads to dephosphorylation of TRPV1 receptors [19]. Since the activation of TRPV1 transiently increases cytosolic Ca^2+^ levels, even low concentrations of agonist can raise cytosolic Ca^2+^ concentrations and desensitize the receptor. This dephosphorylation occurs by enzymes such as calcineurin (protein phosphatase 3) [68], which can dephosphorylate serine and threonine residues that were previously phosphorylated by PKA [69]. This decrease in TRPV1 phosphorylation could then diminish capsaicin channel sensitivity [68] and cause a decrease in response to agonists such as capsaicin. In support of this hypothesis, receptor phosphorylation by PKC has been shown to diminish Ca^2+^-induced desensitization of TRPV1 receptors [68, 69].

Conversely, TRPV1 may exhibit the lowest responses to agonist if the receptor is dephosphorylated prior to agonist (capsaicin) binding. TRPV1 phosphorylation by PKC may mobilize TRPV1 to the plasma membrane from internal (vesicular) membranes [67]. If true, then these results would imply that dephosphorylated receptors localize primarily to internal membranes where TRPV1 receptors might have less access to hydrophobic agonists such as capsaicin. If so, then this internal pool of dephosphorylated (and possibly nontetrameric) receptors might underlie capsaicin desensitization.

TRPV1 receptors are also stabilized by the cytoskeleton [70]. Tubulin dimers directly bind the C-terminus of TRPV1, and this interaction could decrease TRPV1 activity under depolymerizing conditions [71], possibly at high Ca^2+^ concentrations. Finally, capsaicin-sensitive channels can form heterotetramers with either TRPA1 or TRPV3 subunits in heterologous systems [72, 73]. These heterotetramers have channel properties that differ from TRPV1 homotetrameric channels.

### 2.4. Regulation of TRPV1 Channels by Membrane Lipids

TRPV1 is highly sensitive to its membrane environment. For example, this receptor requires membrane cholesterol for optimal channel activity in CHO cells [74]. In addition, TRPV1 channels are highly sensitive to their phospholipid environment [75]. One important modulator of TRPV1 activity is PIP_2_, a membrane phospholipid that directly interacts with TRPV1 channels [74, 76, 77]. PIP_2_ is a physiologically important phospholipid that is formed from phosphatidylinositol and localizes to the inner leaflet of native mammalian plasma membranes [78]. The C-terminal regions of TRPV1 channels between amino acids 777–820 as well as amino acids R701 and K710 near the TRP domain are thought to be responsible for directly interacting with PIP_2_ [31, 76] (see Figure 2(a)).

Activation of the phosphatidylinositol signaling cascade by TRPV1 agonists such as bradykinin will decrease membrane PIP_2_ concentrations (a substrate for PLC*β*) by hydrolyzing this lipid to IP_3_ and DAG. This decrease in membrane PIP_2_ could directly modulate TRPV1 activity in plasma membranes [39, 76]. This modulation may occur by releasing TRPV1 from PIP_2_-dependent inhibition [79], or by PKA-mediated recovery from inactivation [62].

Several lines of evidence suggest that PIP_2_ is a positive modulator of TRPV1 activity [76]. For example, positively charged epsilon amino groups of polylysine sequester anionic membrane lipids by electrostatic interactions with negatively charged phosphoinositide headgroups [80]. The addition of polylysine to TRPV1-containing membranes inhibits capsaicin-activated currents [81].

Several excised inside-out patch clamp studies have shown that inositol phospholipids directly affect TRPV1 channel activity [76]. The application of PIP_2_ to the intracellular leaflet of excised membrane patches, or application of this lipid to one side of a planar membrane containing reconstituted TRPV1 channels, activates TRPV1 channels [75, 77]. PIP_2_-activated TRPV1 also shows a decrease in activity due to the gradual dephosphorylation of PIP_2_ and its precursor phosphatidylinositol 4-phosphate (PI_4_P) by phosphatases in membrane patches [76, 82]. The activity of TRPV1 channels at low capsaicin levels decreased by approximately 90% from initial activity after excision of the membrane patch into an ATP-free medium [75, 76]. TRPV1 channel activity was restored by directly adding Mg^++^ATP to excised patches. This restoration was prevented by adding inhibitors of phosphatidylinositol 4-kinase, or inhibitors to a phosphatidylinositol-specific bacterial phospholipase C enzyme [75, 76]. These results indicate that PIP_2_ sensitizes TRPV1 channels, and the depletion of this membrane lipid may lead to channel desensitization [76].

Membrane PIP_2_ concentrations may be decreased by mechanisms that do not involve the generation of second messengers via PLC. For example, the immunosuppressive compound rapamycin from the bacterium* Streptomyces hygroscopicus* causes the translocation of 5′-phosphatase to the plasma membrane where this enzyme dephosphorylates PIP_2_ to PI_4_P [77]. Studies with this inducible 5′-phosphatase indicated that TRPV1 was not inhibited by rapamycin [77] at high concentrations of capsaicin. These results suggested an important role for PI_4_P in modulating TRPV1 channel activity under these conditions [77]. Taken together, these studies suggest that endogenous inositol phosphates enhance TRPV1 channel activity in excised membrane patches [76, 77, 81, 83, 84] similar to their effect on K channels [85]. Finally, the combined depletion of both PIP_2_ and PI_4_P in native membranes inhibits capsaicin-induced TRPV1 channel activity in these membranes [86].

After initial activation of TRPV1 by capsaicin, high levels of this agonist reduce channel responsiveness in trigeminal neurons [87]. One interpretation is that maximal stimulation of TRPV1 receptors and subsequent Ca^2+^ influx depletes PIP_2_ in membrane bilayers. This decrease in PIP_2_ and its precursor PI_4_P may be caused by hydrolysis of PIP_2_ by Ca^2+^-sensitive isoforms of phospholipase C*δ*. If PIP_2_ stimulates channel activity, then a decrease in PIP_2_ would limit channel activity [88]. TRPV1 channel activity may then be inhibited and possibly undergo desensitization [89]. If true, then degradation of PIP_2_ by PLC isoforms (which hydrolyze PIP_2_ to IP_3_ and DAG) would decrease membrane PIP_2_ concentrations and subsequently limit channel activity during capsaicin-induced desensitization [76].

Alternatively, PIP_2_ may inhibit TRPV1 function [48, 90]. Purified TRPV1 protein that was reconstituted into artificial lipid membranes devoid of phosphoinositides was fully active, which suggested no dependence of TRPV1 channel activity on phosphoinositides [90]. However, the response of purified TRPV1 to both capsaicin and heat decreased when phosphatidylinositol, PIP_2_, or PI_4_P was added to membranes. This finding suggested that membrane phosphoinositides inhibited TRPV1 in reconstituted systems [90]. In addition, recent studies suggest that purified TRPV1 channel activity is inhibited at its C-terminus by PIP_2_ when this phospholipid localizes to both leaflets of the plasma membrane [86]. Finally, a TRPV1 mutant protein lacking a putative PIP_2_ binding site in the C-terminal region suggested that PIP_2_ binding caused an inhibitory effect on channel activity [48, 79].

The contradictory results concerning PIP_2_ modulation of TRPV1 channel activity may be explained by phosphatidylinositol distribution in membranes. In native plasma membranes, PIP_2_ localizes to the inner leaflet of the bilayer where this inositol lipid may positively regulate TRPV1 channel activity [76]. Recent data has suggested that PIP_2_ localized to the inner (cytoplasmic) leaflet activates TRPV1 while PIP_2_ localized to both the outer (extracellular) and inner (cytoplasmic) leaflets inhibited TRPV1 [91]. Patch clamp fluorometry studies with synthetic PIP_2_ further indicated that PIP_2_ must incorporate into the outer leaflet for inhibition to occur [91]. This observation could explain why PIP_2_ inhibited TRPV1 activity in reconstituted membranes where this phospholipid is presumed to be distributed equally between both membrane leaflets [91].

However, PIP_2_ is a lipid component of both plasma membranes and internal membranes. Some evidence suggests that membrane phospholipids are symmetrically distributed between the two leaflets of ER (microsomal) membranes [92]. If membrane asymmetry is required for PIP_2_ to function as a positive regulator of TRPV1, then a symmetric distribution of PIP_2_ in ER membranes could modulate TRPV1 differently from an asymmetric distribution of PIP_2_ (and PI_4_P) in plasma membranes. Nonetheless, these studies do indicate a dependence of TRPV1 channel activity on membrane phosphoinositides. The relative contributions of PIP_2_ and PI_4_P in plasma membranes and internal membranes, and partitioning of TRPV1 in membranes following agonist binding, remain to be determined [76].

### 2.5. Capsaicin Perception in the Human Oral Cavity

The consumption of chili peppers in the human diet dates back to 7000 BC [93]. This consumption resulted in the domestication of these plants approximately 1000 years later in Mexico or northern Central America from wild bird pepper plants [93, 94]. Hot-tasting foods from these plants were subsequently introduced to Europeans when these early explorers returned from the Caribbean [95].

Capsaicin and dihydrocapsaicin are the two major capsaicinoids synthesized by chili peppers, and these two vanilloids are responsible for approximately 90% of the total pungency of chili peppers [96]. Currently, chili peppers are some of the most widely consumed spices in the world [97]. The food industry is the largest consumer of chili peppers, which uses these compounds as flavoring agent in foods, noncarbonated drinks, and alcoholic beverages [98]. Chili peppers can also interact with other foods such as cheese sauces, chicken patties, pork patties, sucrose, sodium chloride, and citric acid [99–102]. Chili peppers may also increase the flavor of vegetables. This flavor enhancement would presumably enhance the ingestion of vegetables and decrease the consumption of fats and cholesterol-rich foods [103].

Due to its dietary importance, capsaicin perception in the oral cavity has been extensively examined in human psychophysical studies. As opposed to taste stimuli, capsaicin is a hydrophobic compound that is difficult to administer to the oral cavity in aqueous solution by “sip and spit” chemosensory assays. Since capsaicin exhibits low solubility in water (0.0013 g/100 mL), psychophysical testing of this stimulus at suprathreshold concentrations is often performed in ethanolic solutions [104], or with impregnated (dried) filter papers [105]. Other delivery methods include the use of rapidly dissolving edible films [106]. Edible film delivery of capsaicin can identify chemosensory properties of this vanilloid in the oral cavity and can demonstrate that a variety of oral stimuli modulate capsaicin chemosensation in the oral cavity [106].

## 3. Psychophysics of Capsaicin Perception

Capsaicin causes a burning sensation and affects the perception of temperature in the oral cavity [107]. Interestingly, the tip of the tongue is thought to be the most responsive region to capsaicin [108]. This burning sensation may alter preferences for this vanilloid in humans. Frequent consumers of chili peppers generally indicate that “hot-tasting” spices enhance the taste of food, while individuals who avoid spicy foods indicate that hot spices reduce or mask food flavors [109]. In some instances, development of preferences for capsaicin in the oral cavity can be associated with an affective (mood) shift from “dislike to like” for hot-tasting foods [110, 111].

In humans, capsaicin-rich foods may produce “gustatory sweating.” This phenomenon causes flushing of the face and the appearance of perspiration on the face and scalp [113]. “Gustatory sweating” is thought to be caused by the effect of capsaicin on the control of thermoregulation [113].

Rats normally avoid diets that contain chili pepper and rarely develop preferences for oral irritants [114]. In 1960, Jancsó reported that systemic application of capsaicin could cause an extended loss in chemical irritant sensitivity in rodents [115], and rats became indifferent to these diets after systemic desensitization to this stimulus [114].

Human studies have demonstrated that topical application of high concentrations of capsaicin to the tongue for ten-minute periods caused a large drop in irritant sensitivity, and this loss of sensitivity did not recover for up to 48 hours [116, 117]. In addition, changes in taste perception from exposure to capsaicin did not occur. However, interactions were predicted to occur between taste receptors and TRP receptors [17, 118].

Exposure to gradually increasing amounts of chili in the human diet appears to be a sufficient condition for developing a preference for spicy foods [110]. Individuals that consume spicy foods begin to enjoy this burning sensation in the oral cavity. The basic change to “liking” for chili peppers is thought to correspond to an affective shift that represents a change in the evaluation of peripheral sensory input [110, 111]. Finally, this hedonic shift toward acceptance may result from an understanding that the burning sensation is harmless and may correspond to the enjoyment of constrained risks [110].

Few studies have examined the effect of temperature on capsaicin perception in the oral cavity [107]. In 1986, Green demonstrated that the perceived intensity of the burning sensation of 2 ppm capsaicin increased in a linear fashion when the solution temperature varied from 34 to 45 degrees Celsius [107]. Tongue surface temperature was not measured, and no conclusions could be drawn as to whether TRPV1 receptors were activated by heat (at a temperature ≥43°C). However, capsaicin can affect sensitivity to pain. Heat stimulation with a Peltier thermode is a standard procedure for examining human sensitivity to pain. The surface temperature of the tongue can be transiently increased with a square Peltier thermode in order to induce oral pain. Capsaicin (33 *μ*M) significantly enhanced pain ratings to heat stimuli in the temperature range of 47°C to 50°C for a period of at least 5 minutes after presentation of this irritant [119]. The authors concluded that capsaicin enhanced the thermal gating of TRPV1 in cells that mediated thermal pain sensation [119].

The effect of primary taste qualities on capsaicin perception in the oral cavity has been examined. Stevens and Lawless [120] reported that all four primary taste qualities (sweet, sour, salty, and bitter) attenuated the burning sensation of capsaicin in the oral cavity. The decline in the intensity of capsaicin perception following an oral rinse with a taste stimulus occurred most rapidly with citric acid (sour taste) and sucrose. Sodium chloride produced less of an attenuating effect on capsaicin, while the bitter taste stimulus quinine had the least effect on capsaicin perception [120]. Capsaicin underwent exponential decay in perceived intensity following oral rinses with primary taste stimuli [120].

Nasrawi and Pangborn [121] reported that room temperature rinses with 10% sucrose or whole milk at 5°C reduced the oral burn of 3 ppm capsaicin. Sizer and Harris [99] further noted that thresholds for capsaicin mouth-burn were suppressed by sucrose, but not by sodium chloride and/or citric acid.

Psychophysical studies have also identified the effect of capsaicin on the perception of primary taste stimuli. Lawless and Stevens [122] reported that oral rinses with capsicum oleoresin (chili extract containing capsaicin and related compounds) decreased perceived intensities of sour taste (citric acid), bitter taste (quinine), and sweet taste (at high concentrations of sucrose). In contrast, they reported no decrease in salty taste following a capsicum rinse. Their results suggested an inhibitory effect of oral chemical irritation on the perception of primary taste qualities, perhaps by recruiting gustatory nerves for carrying irritant information at the expense of gustatory signals [122].

Lawless and Stevens [108] also reported that capsaicin partially masked both taste and olfactory stimuli. These results suggested an inhibitory effect of oral chemical irritants on the perception of sweet, sour, and bitter taste [108, 120] and supported the earlier results of Sizer and Harris [99]. In contrast, Cowart [123] simultaneously presented capsaicin and individual primary taste stimuli and reported that the perceived intensity of primary taste qualities was generally unaffected by oral irritation with capsaicin. Finally, animal studies have shown that capsaicin at 100 *μ*M concentrations reduced the strong preference for sucrose in* TRPV1* ^−/−^ null mice [118].

The ability of capsaicin to variably mask primary taste stimuli may suggest that capsaicin alone does not directly activate the human gustatory system [124]. However, capsaicin (and the trigeminal stimulus menthol) may stimulate taste responses in some individuals. For example, cotton swabs that contain 100 or 320 *μ*M capsaicin elicit a bitter taste response in the human oral cavity, most notably when applied to circumvallate papillae [124]. In addition, a modified TRPV1 channel has been proposed as a candidate Na^+^ taste receptor/channel [125–127].

Overall, these results suggest that primary taste stimuli can partially mask the burning sensation of capsaicin and that capsaicin rinses may also attenuate primary taste stimuli in the oral cavity. These findings suggest that interactions may occur between trigeminal neurons and taste receptor cells in the oral cavity, or that signal integration between trigeminal neurons and the gustatory system might occur in the CNS.

These results may imply that TRPV1 receptors and taste receptors are expressed in the same sensory cell or that trigeminal neurons directly communicate and interact with specific populations of taste receptor cells. However, the expression of TRPV1 in taste receptor cells remains controversial [17, 128]. If coexpression of taste receptors and TRPV1 receptors is conclusively shown to occur within a sensory cell [128], this finding could explain how capsaicin attenuates taste responses that stimulate cytosolic Ca^2+^ flux. For example, sweet or bitter taste stimuli could deplete TRPV1-containing cells of Ca^2+^, a signal that is common to all three cellular transduction pathways. The amount of cytosolic Ca^2+^ could be rate limiting if sensory cells coexpressing TRPV1 and sweet or bitter taste receptors did not activate plasma membrane store-operated channels for subsequent influx of extracellular Ca^2+^. If true, these findings would also argue against the labeled line model for information coding of chemosensory signals in the oral cavity.

### 3.1. Capsaicin Thresholds in the Oral Cavity

The threshold for an oral chemosensory stimulus is defined as the lowest concentration or amount at which a subject is able to detect that stimulus. A variety of oral thresholds have been reported for capsaicin in the human oral cavity. In an early study, Sizer and Harris [99] reported a mean threshold value of 5.9 nanomoles (in 10 mL volumes) for capsaicin. In addition, they reported that thresholds decreased (became more sensitive) as solution temperature increased. Finally, these authors demonstrated that sucrose increased thresholds for capsaicin when both stimuli were presented simultaneously [99].

When investigating concentrations between 0.06 and 4.00 parts per million (ppm) in 0.5 log_2_ unit steps, Rozin et al. [129] reported a threshold of approximately 0.310 ppm in aqueous solutions (5.1 nmol in 5 mL volume). Lawless et al. [130] compared capsaicin thresholds in aqueous solutions and oil-based delivery methods. The concentration of capsaicin in water varied from 0.03125 to 0.500 ppm. The mean threshold was also 0.310 (±0.03) ppm in aqueous solution, which is consistent with the results of Rozin et al. [129]. The findings of Rozin et al. [129] and Lawless et al. [130] generally agreed with the results of Sizer and Harris [99], who reported threshold values between 0.090 and 0.350 ppm (2.9 nmol to 11.5 nmol in 10 mL volumes). Karrer and Bartoshuk [131] reported that a concentration of 0.100 ppm did not induce a response in all test subjects. However, Green [132] used capsaicin-impregnated filter paper disks as the delivery method and identified mean thresholds near 0.09–0.10 ppm. Finally, Rentmeister-Bryant and Green [133] reported mean thresholds of 0.299 ppm for capsaicin on the tongue.

More recent studies have reported absolute thresholds of 0.050 ppm (1.6 nmol) for total capsaicinoids, where total capsaicinoid content was taken as the sum of capsaicin and dihydrocapsaicin in the test sample [134]. In these studies, detection thresholds for capsaicin and dihydrocapsaicin in aqueous solutions, or emulsified with polysorbate 80, were identified by a three-alternative forced-choice method. Mean thresholds were near 0.080 ppm (1.3 nmol). In this study, “users” and “nonusers” of chili did not differ significantly in their perception of capsaicin [134]. Finally, Smutzer et al. [106] reported mean recognition thresholds for capsaicin near 1 nmol when the stimulus was delivered by pullulan-based edible strips. A possible explanation for these variations in thresholds could be due to the placement of stimulus on different regions of the tongue surface. Variations in thresholds could also be explained by the different methods that were used for stimulus delivery. Finally, diet could affect capsaicin thresholds since diets rich in spicy foods could cause chronic desensitization in some individuals [132]. Nonetheless, these thresholds are considerably lower (more sensitive) than mean thresholds for bitter taste stimuli [135], including* n*-propylthiouracil (PROP) [136].

In humans, thresholds to oral chemosensory stimuli may be affected by anxiety levels [135]. Behavioral factors may also affect responses to trigeminal stimuli. In particular, personality factors may drive differences in the liking and consumption of spicy foods that contain capsaicin [137]. These affective responses may influence responses to the previously aversive burning sensation of capsaicin.

Finally, some evidence suggests that the pleasure obtained from the irritating qualities of capsaicin in the oral cavity can be learned following repeated exposure to this irritant [110, 138]. This increased pleasure may be caused by repeated exposure to a cuisine rich in spicy foods [139]. In addition to genetic and cultural factors, human personality traits may have an important role in determining hedonic responses to capsaicin-containing spicy foods [140].

### 3.2. Capsaicin Sensitization

Sensitization is caused by an increase in perceived intensity following the repeated exposure of that irritant at short stimulus intervals [132, 141–144]. Psychophysical studies have shown that sensitization has been observed following either oral or cutaneous application of capsaicin [132]. Also, test subjects show variation in sensitization patterns to capsaicin in the oral cavity [145]. However, not all chemosensory stimuli cause sensitization. For example, repeated stimulation with odors generally shows a reduction in stimulus intensity if the interstimulus interval is brief [146].

In psychophysical studies, low (3 ppm) concentrations of capsaicin that do not elicit oral pain produce an increase in perception (piquancy) during repeated stimulation with interstimulus intervals between 30 and 60 seconds [132, 145]. In the human oral cavity, repeated stimulation with capsaicin resulted in a monotonic increase in perceived intensity. This sensitization occurred after only a few applications of stimulus [132] and continued for up to 25 sequential exposures when no oral rinse was presented between exposures [132]. In this study, filter papers saturated with 3 ppm capsaicin were presented at one-minute intervals. This procedure caused more than a doubling in rating intensity for capsaicin following ten successive applications of irritant [132]. Capsaicin sensitization may have occurred from an increase in stimulus concentration at the site of application on the tongue surface, rather than from spatial summation from converging neurons [147]. However, the mobilization of TRPV1 receptor to the plasma membrane, formation of functional homotetrameric channels, interactions with inositol phospholipids, or receptor phosphorylation could also underlie sensitization.

Capsaicin sensitization in the human oral cavity may be independent of the delivery method since filter paper applications and whole mouth rinses composed of 0.6 or 3 ppm capsaicin in 5% ethanol yielded similar increases in perception of this irritant [145]. However, trigeminal irritants such as zingerone (which may also activate TRPV1 receptors [28]) and menthol (a TRPM8 agonist) show little or no sensitization in the human oral cavity [147–149]. Rather, zingerone induces a progressive decline in irritant intensity after repeated stimulation in the majority of test subjects [147, 148].

Secondly, cross-sensitization of TRP channels could contribute to an enhanced sensitivity to TRPV1 agonists [150]. The sensitization of TRPV1 may occur via activation of TRPA1 receptors in cells that express both cation channels [151]. Since 30% of TRPV1-expressing nociceptive neurons also express TRPA1 channels [152], TRPV1 sensitization could be modulated by TRPA1. In the oral cavity, sensitization of TRPV1 could also involve the activation of adenylyl cyclase, increased cyclic AMP levels, the subsequent translocation and activation of PKA, and phosphorylation of TRPV1 at PKA phosphorylation residues. A possible role for regulatory proteins such as Tmem100 in modulating TRPA1-TRPV1 interactions [153] in oral trigeminal neurons remains to be explored.

Thirdly, low sodium levels may lead to capsaicin sensitization [61]. The lowering of external sodium concentrations directly activates TRPV1 channels in heterologous expression systems [61]. The lowering of external cellular sodium levels could directly gate and sensitize human TRPV1 channels by allowing a greater influx of Ca^2+^, a cation that could be rate limiting under physiological sodium concentrations [61].

Finally, sensitization may occur by mobilizing dimeric capsaicin receptors into functional homotetramers. Since TRPV1 dimers bind ligand before forming functional tetrameric receptors [53], sensitization may be associated with mobilization of functional tetrameric receptors in neuronal membranes.

### 3.3. Capsaicin Desensitization

Desensitization is defined as the diminished sensory response to an aversive stimulus after repeated (discontinuous) exposure to that stimulus. Capsaicin desensitization differs from sensory adaptation because a delay of several minutes is required for capsaicin desensitization to occur [154]. Desensitization may be the result of strong or repeated activation of nociceptors that in turn cause a loss in sensitivity to an irritant [150]. Two different types of desensitization have been associated with TRPV1 receptors. One type is classified as acute desensitization, which is a rapid loss of receptor activity during agonist binding. Acute desensitization of TRPV1 is caused by agonist-induced conformational changes, which close TRPV1 channel pores [31]. This pore closing is dependent on cytosolic Ca^2+^ levels [16]. The second type of desensitization is tachyphylaxis, which is identified as a gradually diminishing response of agonist following repeated administrations of that agonist [14, 31]. Capsaicin-induced tachyphylaxis has been widely examined in the human oral cavity [105]. In general, tachyphylaxis may involve the cycling of TRPV1 channels between resting and active states [28].

In the oral cavity, repeated exposure to capsaicin at identical concentrations causes an increase in perceived intensity. Following an interval of 15 minutes, the sensation produced by capsaicin is less than that of the initial application of capsaicin [141]. Thus, the initial burning sensation of capsaicin in the mouth is generally followed by an extended refractory period [82, 155]. Following an extended time period, the reapplication of capsaicin to the tongue produces a diminished intensity that is identified as self-desensitization [131]. Intervals as short as 5 to 15 minutes can decrease the intensity of subsequent capsaicin applications in the oral cavity. In humans, capsaicin induces desensitization [150, 156], and this property is thought to give capsaicin its anesthesia-like properties.

Under certain conditions, repeated exposure to capsaicin and spicy foods in the diet can cause chronic desensitization in humans [109, 131, 157]. Chronic desensitization may in part be responsible for variations in capsaicin perception in the oral cavity among test subjects in psychophysical studies that are described below [158].

Rozin and Schiller [110] identified capsaicin thresholds and salivation volumes and found a small desensitization effect from capsaicin in chili peppers that were consumed at moderate amounts by test subjects. They reported a modest increase in capsaicin thresholds in subjects who liked chili peppers. These authors also reported that liking of the orally irritating qualities of capsaicin could be learned with repeated exposure to this vanilloid in the human diet [110, 159].

In 1989, Green [132] reported that varying the amount of time between applications of capsaicin produced either sensitization or desensitization. Desensitization could occur with as few as five oral applications of stimulus, as long as a minimum delay of 2.5 to 5 minutes between applications was provided [131]. This capsaicin desensitization could last for several days [131].

Nasrawi and Pangborn [160] demonstrated that the intensity of mouth-burn from repeated stimulation with capsaicin did not increase in an additive fashion for all subjects. They further reported that “eaters and noneaters” of chili pepper showed no differences in their perception of capsaicin. However, capsaicin desensitization may be associated with diet where regular users of chili have reported desensitization to the perception of capsaicin-containing foods [132, 161]. Finally, chronic capsaicin desensitization in the oral cavity may be caused by personality differences that drive a wide range of hedonic responses and subsequent consumption of spicy foods [137].

Capsaicin also desensitizes bitter taste [104]. Desensitization with 100 ppm capsaicin decreased the perceived bitterness of several taste stimuli [131]. Pretreatment with capsaicin desensitized the lingual epithelium and reduced the perception of bitter stimuli that included quinine HCl, urea, and PROP [162].

### 3.4. Long-Lasting (Chronic) Desensitization to Capsaicin

An extended exposure to capsaicin may cause long-lasting desensitization to this stimulus [163]. For example, desensitization to capsaicin increases as exposure to this irritant occurs over a span of weeks [164]. The mechanism for long-term desensitization has been explained as an excessive influx of cations and anions across the plasma membrane of capsaicin-sensitive cells [163]. This ion influx is thought to cause a progressive fatigue-like process in sensory neurons of the oral cavity [154]. However, these conclusions do not agree with more recent studies where desensitization could be produced by rapid and repeated topical exposure to this stimulus, followed by an interval where exposure was stopped for several minutes and then resumed [154].

However, intensity responses to oral capsaicin do vary in humans [165]. This variation could be explained by the frequency of exposure to capsaicin in the diet. For example, intensity responses to capsaicin could decrease over time as dietary consumption of this vanilloid increases [109, 123] or becomes an integral component of the diet. This increased (or continued) consumption could in turn lead to long-lasting desensitization to oral capsaicin.

### 3.5. Desensitization at the Receptor Level

For TRPV1, desensitization may be mediated by Ca^2+^ influx, which leads to a loss of sensitivity to capsaicin [68, 166]. Calcium ions activate calmodulin, which then stimulates calcineurin-mediated dephosphorylation and desensitization of TRPV1 channels [49]. Calcineurin causes capsaicin-induced desensitization by dephosphorylating amino acids that were previously phosphorylated by PKA [55].

After exposure to high capsaicin concentrations, an increase in cytosolic Ca^2+^ levels may activate PLC*δ* [76], an enzyme that hydrolyzes PIP_2_ to DAG and IP_3_. This hydrolysis depletes PIP_2_ (and PI_4_P) levels in the inner leaflet of plasma membranes, which in turn likely decreases TRPV1 channel activity in native membranes [76, 89, 92]. This depletion could limit TRPV1 channel activity and lead to desensitization [77]. A possible role for disassembly of tetrameric TRPV1 channels into dimeric complexes as a model for channel desensitization has not been examined. Finally, desensitization of TRPV1 channels may be caused by a decrease in affinity for capsaicin since receptor responses can be recovered by raising capsaicin concentrations [167].

Following a protracted exposure to capsaicin, TRPV1 activity may decrease by desensitization (self-desensitization) [132, 168]. Desensitization of TRPV1 may produce the analgesic effect of capsaicin [49]. Extracellular Ca^2+^ ions are required for channel desensitization because Ca^2+^ influx and release of intracellular Ca^2+^ stores mediate this effect [169]. Calcium influx leads to a loss of sensitivity to capsaicin along with a reduction in heat sensitivity [166]. Also, desensitization of TRPV1 by capsaicin decreases the apparent affinity for this agonist since responses can be recovered by raising capsaicin concentrations [167].

At the molecular level, long-lasting desensitization to capsaicin could be caused by a decrease in functional TRPV1 receptors in sensory neurons, lack of mobilization of functional receptors to the plasma membrane, a decrease in kinase activity in these cells, or changes in membrane lipid interactions with this receptor. An explanation for long-term desensitization to capsaicin in the oral cavity, and the possible role of diet in this phenomenon, awaits further study.

### 3.6. Stimulus Induced Recovery following Desensitization

The repeated application of capsaicin to previously desensitized oral epithelium induces irritation that approaches the original perceived intensity of capsaicin [105, 154, 170]. These results indicate that desensitization can be temporarily reversed if oral stimulation with a trigeminal stimulant such as capsaicin is resumed. This response to desensitized tissue has been named stimulus induced recovery (SIR). SIR occurs when lingual application of capsaicin induces the activation of TRPV1 receptors, and this activation overcomes prior desensitization by this stimulus [105, 154, 170].

SIR is caused by rapid exposure to capsaicin in concentrations at least as high as the desensitizing stimulus, and at short interstimulus intervals [133]. This reapplication of stimulus may result in maximal intensities that were previously observed during sensitization with agonist [105, 154]. These findings suggest that capsaicin desensitization could be reversed by further application of this irritant and that desensitization and SIR were likely facilitated by opposing cellular (or TRPV1 receptor) processes.

At the receptor level, SIR may be the result of mobilization of low-affinity TRPV1 receptors that were not desensitized during initial applications of capsaicin [170]. Therefore, SIR may involve cellular mechanisms similar or identical to those that trigger capsaicin sensitization [170] (i.e., low-affinity receptors that failed to form homotetramers). Finally, some evidence suggests that PIP_2_ synthesis from PI_4_P by PI_4_P kinase is required for the recovery of TRPV1 channels from prior desensitization [171].

Interestingly, SIR is not unique to capsaicin. The trigeminal stimulant piperine (a component of black pepper) is a vanilloid that also induces SIR under conditions of self-sensitization, or by cross-desensitization with capsaicin [154]. Both capsaicin and piperine are TRPV1 agonists [172].

Zingerone (from ginger) is primarily a TRPA1 agonist [173] that does not exhibit SIR [154]. However, zingerone can activate TRPV1 channels in heterologous systems where inositol phospholipids may be uniformly distributed in both leaflets of the bilayer [27]. Menthol is also a trigeminal stimulus (counterirritant) and a TRPM8 agonist that does not induce SIR when capsaicin is the stimulus [149]. However, menthol does show transient desensitization (cross-desensitization) of capsaicin irritation in the oral cavity [174].

SIR may occur from interacting factors at the cellular level or may be the result of higher order neuronal processing. However, these results do suggest a functional role for TRPV1 receptors in SIR. As previously mentioned, PIP_2_ enhances TRPV1 activity in native membranes. At the present time, a possible role for membrane PIP_2_ levels (or receptor phosphorylation) in SIR has not been explored. However, activation of PKC reverses the capsaicin-induced desensitization of TRPV1 channels [65], which could explain the underlying mechanism of SIR.

## 4. Conclusions

In summary, capsaicin is a naturally occurring compound that causes a pungent sensation in the human oral cavity when this vanilloid binds to TRPV1 receptors. Functional TRPV1 receptors form homotetramers in biological membranes, are highly regulated in neuronal tissue, and are highly sensitive to their membrane lipid environment. Along with receptor phosphorylation, recent data have suggested that inositol lipids such as PIP_2_ and PI_4_P stimulate this receptor in native membranes. TRPV1 mobilization to the plasma membrane and the effects of receptor phosphorylation and TRPV1 interactions with inositol phospholipids on capsaicin perception in the oral cavity remain largely unexplored.

Human psychophysical studies have indicated that capsaicin may modulate the perception of primary taste stimuli and that some primary taste stimuli may in turn modulate capsaicin perception. Recent psychophysical studies have further indicated that capsaicin causes sensitization, desensitization, and SIR in the oral cavity. Further studies are required to determine possible interactions between TRPV1-expressing cells and taste receptor cells in the human oral cavity. Future studies are also needed to more fully integrate molecular studies with psychophysical studies and to clarify possible interactions involving signals from primary taste stimuli and trigeminal irritants in the CNS. Finally, the possible role of TRPV1 single nucleotide polymorphisms in modulating vanilloid perception in the oral cavity remains largely unexplored [175]. The results of these studies will serve to clarify the effects of capsaicin on the human diet, and its role as an analgesic for treating pain.
